# Aspects of the Immunopathogenesis of Lyme Arthritis

**DOI:** 10.3390/microorganisms13071602

**Published:** 2025-07-07

**Authors:** Leonard H. Sigal

**Affiliations:** Gateway Immunosciences, Stockbridge, MA 01262, USA; lensigal@gmail.com

**Keywords:** Lyme disease, Lyme arthritis, *Borrelia burgdorferi*

## Abstract

Lyme disease has many different manifestations, including arthritis. There is no evidence that the pathogen *Borrelia burgdorferi* expresses toxins, so the presence of the organism is insufficient to cause inflammation and disease. Thus, the interaction between the causative pathogen and the many human immune system mechanisms responding to the organism is the apparent cause of inflammation and tissue damage/dysfunction. This review discusses many of the aspects of the relationship between organisms and host responses to summarize the many means by which the immune response can cause the synovitis of Lyme arthritis.

## 1. Introduction

“Lyme disease” (LD) was first reported by an investigation of a cluster of patients with mono-arthritis initially described as being juvenile rheumatoid arthritis (JRA) (now “juvenile idiopathic arthritis” [JIA]) in three towns along the east bank of the Connecticut River [1]. However, as the researchers from Yale noted, JRA does not occur in clusters—not geographic, familial, or temporal. The distribution of cases suggested an arthropod-borne infection [2,3], although no pathogen was immediately identified. Many of these patients recalled a tick bite and/or an expanding erythematous rash, consistent with a rash initially described in Sweden by Afzelius in 1909 [4], first noted in the United States in 1970 by Scrimenti in Wisconsin [5]; it was called erythema chronicum migrans (ECM) [3] and was subsequently named erythema migrans (EM).

Residents of eastern Long Island, NY, and the New England coast may have experienced Lyme arthritis (LA) many years earlier, with the occurrence of “Montauk knee” reported in the 19th century [6]. Lyme arthritis was described in the US but may have been present in Europe millennia before the reports of EM or arthritis. Arthritis is only one of the many musculoskeletal consequences of this infection [6,7].

The clinical spectrum of LD soon expanded [2,6,7,8,9] with the identification of other infection-linked skin lesions, as well as a variety of neurologic and cardiac features. Antibiotics were curative for European EM, suggesting an infectious cause; later, intravenous penicillin was shown to be effective in most patients with Lyme arthritis [10,11,12,13]. Ultimately, *Borrelia burgdorferi* was identified as the cause of LD [14]. There are several similar organisms causing LD around the world with different characteristics, although a full discussion of this is beyond the scope of this review. These include [15].


**NAME**

**DISEASE ASSOCIATION**

**DISTRIBUTION**

*B. afzelii*
implicated Asia, Europe
*B. bavariensis*
implicatedAsia, Europe
*B. bissettiae*
possibleEurope, North America*B. burdorferi*, sensu strictoimplicatedEurope, North America
*B. garinii*
implicatedAsia, Europe
*B. kurtenbachii*
implicatedNorth America
*B. lusitaniae*
possibleEurope
*B. mayonii*
implicatedNorth America
*B. spielmanii*
implicatedEurope
*B. yangtzensis*
possibleAsia
*B. miyamotoi*
*B. miyamotoi* diseaseAsia, Europe, North America

There are more members of the *B. burgdorferi* sensu lato family not yet implicated in disease, with the possibility of more to be found in the future.

European Lyme borreliosis and US LD are similar, with differences in the pathogen and the immunogenetics of patients possibly contributing to the differences in clinical features [8,16,17,18,19,20].

*B. burgdorferi* employs several ways to escape the inoculation site and disseminate [7,9,19,20,21,22,23]. The reviews by Coburn et al. and Smith et al. describe the many factors produced by *B. burgdorferi* to enter the host, survive, and evade host immune mechanisms [18]. Bockenstedt and Wormser have published a concise review of LD [24]; Bockenstedt and Belperron have published a review of how omics studies are assisting in our understanding of Lyme disease [25].

**Hypothesized pathogenesis of these clinical manifestations**

Local presence of organism, dead or alive;
Pro-inflammatory molecule release;
Autonomous self-perpetuating immune/inflammatory reaction;
Molecular mimicry.

## 2. Arthralgias/Migratory Polyarthralgias/Myalgias

Arthralgias—joint pain without inflammation—is common in early LD [10], although true inflammatory joint disease is far less common. The cause of the typically migratory polyarthralgias (noninflammatory) noted by many patients with early LD is unclear, but no evidence has been found of live *B. burgdorferi* within transiently affected joints.

## 3. Mono-Arthritis/Oligoarthritis

Monoarthritis usually affects a single knee and occurs after the dissemination of the organism from the EM, which is the site of inoculation due to the invasion of the vascular or lymphatic endothelium. *B. burgdorferi* can bind fibronectin, decorin, collagen, heparin, and glycosaminoglycans [18,23] in the extracellular matrix and expresses multiple virulence factors predisposing to dissemination, survival, immunogenicity, and escape from host immune responses [18,23,24,25,26].

There is usually remarkable knee swelling, with effusions of over 100 cc common. The knee is stiffer and more difficult to move rather than painful. Many patients do not recall any prior features of LD. IgG anti-*B. burgdorferi* seroreactivity should be present with Lyme arthritis—if the test is negative, the diagnosis of Lyme arthritis should be questioned.

Although the knee is the most commonly affected joint, any joint(s) may be involved, e.g., the shoulder, ankle, elbow, and wrist; arthritis in the small joints of the hands and feet, mimicking rheumatoid arthritis, is unusual. Some patients with LD have persistence of some or all of their initial symptoms or new even new ones, which may include diffuse musculoskeletal pain [27], despite adequate antibiotic therapy.

The interaction of *B. burgdorferi* with host immune systems is complex. *B. burgdorferi* does not bear, produce, or release toxins. Clinical disease is due to the host’s immune response to the organism, with the release of pro-inflammatory molecules as part of the response.

Non-specific responses to *B. burgdorferi* mediated by the innate immune system, reviewed by Brouwer et al. [28], include complement fixation, the C-reactive protein or other scavengers binding to the organism; engagement by Toll-like receptors (TLRs) or nucleotide-binding oligomerization domains (NODs); or recognition by other innate immune receptors. Borrelial antigens are taken up by antigen-presenting cells (APCs), resulting in cytokine release, including interleukins (IL) IL-6, IL-12, IL-17, IL-18, and IL-23 and chemokines (e.g., for Th1 cells—CXCL10 and CXCR3; for Th-17 cells—CXCR3). APCs elicit antigen-specific T cell activation. B cells bind borrelial antigens directly (and can take up these antigens to act as potent APCs). When activated, they can produce pro- and anti-inflammatory cytokines and, of course, antigen-specific antibodies. Recently, fibroblast-like synoviocytes were found to be competent APCs in Lyme synovitis [29]. Both antigen-specific antibodies and antigen-reactive mononuclear cells are concentrated within the synovial space [30]. The immune response in Lyme disease has recently been reviewed in depth [31]. Tokarz et al. used high-density peptide arrays to perform precise epitope mapping for a large number of *B. burgdorferi* antigens. Combining these data with a machine learning approach identified targets in early disease, as well as patterns associated with different manifestations of Lyme disease [32].

Multiple pathways underlie infection causing local and systemic features of disease by the following:

Antigen-specific immune responses with a direct attack on organisms or debris eliciting inflammation;
Innate immune system responses (to “danger-associated molecular patterns” [DAMPs]”, pathogen-associated molecular patterns” [PAMPs]) focusing on live, dead, or dying organisms;
Toxins released by an organism, e.g., *Clostridium difficile* and *Staphylococcus aureus*, although no toxin has been identified from *B. burgdorferi*;
The production of pro-inflammatory substances during the immune response that may effect changes locally or at a distance;
Poorly regulated local immune reactions with a shift to autonomous, independent, and unregulated inflammatory condition;
Poorly regulated immune responses to *B. burgdorferi* causing auto-immune reactivity, e.g., molecular mimicry, to local self-antigen(s).

**Local presence of organisms, dead or alive**

There is ample evidence that *B. burgdorferi* is (or was) present at the sites of inflammation and organ dysfunction in LD, including the skin, heart, brain, and joints. The organism arrives via hematogenous or trans-lymphatic spread from the initial tick bite. Active and viable spirochetes can be found in EM lesion biopsies but not in synovial fluid, despite PCR detection of the organism’s DNA [33], but this does not mean there are no viable organisms bound to synovial tissue. BbHtrA (*B. burgdorferi* high temperature requirement A; 48 kDa), a surface-exposed immunogenic protease found in Lyme arthritis synovial fluid, is a potential partial contributor to its synovial tropism. The organism also expresses aggrecanase activity [34]. It is possible that persisting non-viable organisms that shed antigens (e.g., peptidoglycan) [35] may elicit persisting inflammation in the absence of viable organisms. The persistence of peptidoglycan fragments within joint tissues may contribute to immunopathogenesis, even after appropriate antibiotic treatment. *B. burgdorferi* releases peptidoglycan turnover products; an enzyme now called MltS causes changes that suppress the molecule’s ability to activate NOD2, thereby decreasing the activation of the innate immune system [36]. In the macaque model of LD, “…persisting chronic LD symptomatology might be attributable to residual inflammation due to a low burden of residual antigen and possibly ‘persistent host-adapted spirochetes” [37]. Methyl-accepting chemotactic proteins (MCPs), notably MCP5, regulated by both Rrp1 and Rrp2 pathways, play a critical role in the establishment of infection in mammalian hosts, allowing for the evasion of NK cell-mediated host innate immunity. MCP5 also plays a role in the transmission of spirochetes from ticks to mammalian hosts [38].

Local organism-targeted immune reactivity probably causes inflammation and organ dysfunction; there is no proof of organ-specific autoimmunity in LD. There has never been an isolate of *B. burgdorferi* resistant to the standard antibiotics recommended in the Infectious Disease Society of America (IDSA) guidelines [39], so the prolongation of therapy and/or a shift to agents with a different antimicrobial mechanism is unlikely to be helpful. The only toxic substances present at the site of inflammation are those produced by the host’s immune response.

Inflammation elicited by debris might persist after antibiotic therapy until the persisting antigens of *B. burgdorferi* are cleared. McClune et al., using a murine model, found persistence of *B. burgdoferi*’s peptidoglycan in the liver (both Kupffer cells, capable of antigen presentation, and hepatocytes). Unlike most bacterial peptidoglycan, *B. burgdorferi*’s has a unique polymeric structure that causes murine liver accumulation persisting for weeks but causing minimal liver damage. The secreted protein patterns are similar to those produced in patients with chronic manifestations following acute infection [40]. The persistence of antigens near cartilage was identified in a murine LD model by Bockenstedt et al. following antibiotic treatment, although there were no viable spirochetes identified. The remaining spirochetal components elicited an IgG anti-*B. burgdorferi* antibody response when injected into naïve mice [41]. The “amber hypothesis” is that “non-viable spirochetes or more likely spirochetal debris enmeshed in a host-derived fibrinous or collagenous matrix” can be as the focus of prolonged inflammation [42].

There has been speculation that live dormant *B. burgdorferi* remain in the synovial space, thereby causing ongoing inflammation. These “L-forms” are theorized to be resistant to antibiotics but capable of reverting to an infectious morphology. So-called “persisters” [32] have been produced in vitro but never identified in vivo. Their relevance to the pathogenesis of LD is unproven; presumably, these forms would be targeted by the immune response and if reverted to a “normal” infectious form, they would be susceptible to antibiotics. Intracellular L-forms would be susceptible to the usual intracellular defenses. The organism employs potent defenses to blunt complement fixation [43], suggesting an extracellular existence. Within the phagolysosomal compartment, *B. burgdorferi* is a potent inducer of cytosolic signals resulting in the production of NF-κ B-dependent cytokines, inflammasome assembly with caspase-1 activation, and the induction of programmed cell death [44]. Thus, *B. burgdorferi* employs multiple complex mechanisms to subvert phagocytic, antibody, and complement-mediated destruction, suggesting an extracellular presence [19,43].

**Pro-inflammatory molecule release**

Many of LD’s clinical features can be explained by innate and acquired immune responses [7,16,18,45], e.g., the release of cytokines like IL-1 and tumor necrosis factor-α (TNF-α) and other pro-inflammatory molecules, which can cause fatigue, cognitive dysfunction, and musculoskeletal symptoms [27]. Recent reports have documented the release of type I and type II interferons (IFNs), the former via an interaction of *B. burgdorferi* with TLRs 7 and 9. *B. burgdorferi* is identified by TLRs 7 and 9 to cause peripheral blood mononuclear cell (PBMC) production of NF-κB-associated cytokines and chemokines, including TNF-α, IL-1, IL-6, IL-8, IL-10, and IL-12 [36,38,46]. IL-10 alters effectors induced by *B. burgdorferi* in murine J774 macrophages, suggesting that IL-10 may control inflammation in LD [47]. In a mouse model, neither IL-4 nor IL-13Th2 responses were found to influence the control of spirochetal growth or the intensity of arthritis [40]. Genetic factors in mice determine cytokine production (notably IL-10 and IFN-γ) and susceptibility to arthritis [48,49]. Interestingly, osteopontin levels are decreased in Lyme arthritis, with allelic differences in susceptible and resistant strains of mice [50]. Anti-murine IFN-γ antibodies, when added to cultures of murine lymph node cells, increased the production of anti-borreliacidal antibody by eight-fold; these same borreliacidal antibody-producing cells when infused into C3H/HeJ mice induced severe destructive arthritis after the mice were challenged with *B. burgdorferi* [51].

The type of T cell response strongly influences the clinical consequences of infection. Patients infected with *B. burgdorferi* strain 16s–23s rRNA intergenic spacer type (RST) genotype 1 with the TLR1 polymorphism (TLR1-1805GG) have a stronger Th1-like inflammatory response, suggesting an increased susceptibility to antibiotic-refractory arthritis [52].

On the contrary, a heterozygous human TLR2 (Arg753Gln) polymorphism was associated with a more robust TNF-α and IFN-γ response and was associated with a lesser likelihood of developing late-stage LD [53]. TLR7 and TLR9 recognize *B. burgdorferi* inducing a type I IFN response [38,54]. The production of Type I IFN under the control of a genetic locus termed “Bbaa1” may play a role in the immunopathogenesis of both murine Lyme arthritis and K/B3N serum transfer arthritis (a murine model of RA) [55].

NapA (neutrophil-activating protein A; 22 kDa) is a peptidoglycan-associated protein released from *B. burgdorferi* in outer surface membrane vesicles. It is a virulence factor binding to TLR2 on monocytes, causing the release of IL-1, IL-6, IL-17, IL-23, and transforming growth factor β (TGFβ). NapA is a potent Th17 cell activator, a T cell subset implicated in the persistence of inflammation in rheumatoid arthritis (RA) [56,57,58]. IL-17 released by Th17 cells induces stromal cells, synoviocytes, chondrocytes, fibroblasts, and macrophages to release cytokines; IL-17 is also a potent recruiter and activator of neutrophils [59]. Th17 cells may play roles in helping to control infection, but later, an excess of Th-17 contributes to damage in antibiotic-refractory arthritis [60]. IL-17 and IL-10 are balanced in murine Lyme arthritis; the exogenous anti-IL-17 antibody can suppress arthritis [61]. *B. burgdorferi* stimulates CD14+ monocytes/macrophages (and PBMCs) to secrete the chemokine CCL4, while a mixed PBMC population is required to induce CD14+ cells to secrete chemokines CCL2, CXCL9, and CXCL10 [62].

In a CD14 deficiency murine Lyme arthritis model, there was a decreased activation of MMP 9 and reduced degradation of collagen, as well as a diminished recruitment of neutrophils [63]. The infected tissues of these mice had a higher pathogen burden. CD14^−/−^ macrophages mounted a more severe and persistent inflammatory response due to their inability to be tolerized by *B. burgdorferi*, suggesting that CD14-independent mechanisms may be more potent inducers of inflammation than those incorporating CD14 pathways. This suggests that the efficiency of the neutrophil-mediated clearance of *B. burgdorferi* (here, via the CD14 activation of myeloid cells) may also help determine the severity of the synovitis. Defensins derived from neutrophils (human neutrophil peptide [HNP 1–3]) were identified by Melicherčík et al. in the Lyme arthritis synovial fluid of patients, another indication of neutrophil activity at the local site of infection; the authors suggest these levels might be useful in diagnosis, where other markers are absent [64].

Macrophages are a crucial component of innate immune mechanisms in *B. burgdorferi* infection [65]. Hamsters infused with macrophages exposed to live organisms and enriched populations of immune or naive T lymphocytes develop a more fulminant arthritis after infection with *B. burgdorferi* than recipients infused with either cell type alone, suggesting macrophages and T lymphocytes synergistically induce severe destructive Lyme arthritis [66].

Natural killer (NK) cells and NK T cells both play a role in host defenses early in LD [56]. However, the persistence of large numbers of these cells may play a role in the chronic inflammation and tissue damage of patients with antibiotic-refractory LD [67,68]. IFN-γ induces the differentiation of synoviocytes [69] and suppresses repair mechanisms [70] and has been implicated in chronic synovitis. Mice deficient in MKK3, an upstream activator of p38 mitogen-activated protein (MAP) kinase, have an attenuated Th1 response and production of pro-inflammatory cytokines upon infection with *B. burgdorferi*. The production of IFN-γ by Th1 cells is regulated by tp38 MAP kinase, as are TCR engagement and the IL-12 induction of Th1 cytokines. The direct inhibition of p38 MAP kinase in T cells or administration of an inhibitor of the kinase during murine infection caused a reduction in the serum levels of IFN-γ. The inhibition of this pathway suppressed the production of IFN-γ after anti-CD3 and IL-12 stimulation of *B. burgdorferi*-specific T cells, suggesting that p38 MAP kinase plays a role with *B. burgdorferi*-specific T cells [71]. Thus, there is ample evidence of *B. burgdorferi*’s ability to activate innate immune mechanisms that drive inflammation.

Antigen-specific activated Vα14i NK-T cells recognize *B. burgdorferi* galactosyl diacylglycerol antigens and are important in preventing arthritis in BALB/c mice and enhancing the clearance of *B. burgdorferi*, suggesting that more than merely humoral responses may be needed for resolution [72].

Many components of *B. burgdorferi* elicit antigen-specific immune responses, including surface glycolipids cholesteryl 6-O-acyl-β-D-galactopyranoside (acylated cholesteryl galactoside [ACG or BbGL-I]) and 1,2-di-O-acyl-3-Oα-D-galactopyranosyl-sn-glycerol (monogalactosyl diacylglycerol [MgalD or BbGL-II]), together constituting 36% of the total lipid mass of *B. burgdorferi.* These are highly and persistently immunogenic, suggesting they might be vaccine candidates [73]. A galactoglycerolipid has been identified as a key inflammatory trigger in LD, signaling through TLR2 [74]. Nearly all the 75 Lyme arthritis patients studied had high levels of IgG to both ACG/BbGL-I and MgalD/BbGL-II, whereas a proportion of EM and neuroborreliosis patients had this reactivity, although the role, if any, in the immunopathogenesis of LD is not yet clear [73]. *B. burgdorferi* also causes “polyclonal B cell activation”, which is the non-antigen-specific activation of many B cells [64]; there has been no evidence to implicate this phenomenon in the immunopathogenesis of LD. More recently, dysregulated immune responses impair long-term immunity, with compromised B cell memory and antibody responses having been found [75]. *B. burgdorferi* does not contain a T cell superantigen.

Lochhead et al. described microRNAs (miR) in Lyme arthritis synovial fluid, both before and after therapy. MiR-223 was present during active infection; following antibiotics, the fluid contained inflammatory (miR-146a and miR-155), wound repair (mi-142), and proliferative (miR-17-92) miRs, and levels increased with the duration of arthritis. These miRs were also present in the synovial tissue of those with antibiotic-refractory arthritis, a pattern similar to that seen in rheumatoid arthritis (RA) [76]. MiR-155 may play a role in the pathogenesis of both LD arthritis and carditis. MiR-155 expression is suppressed by IL-10, a potent anti-inflammatory cytokine; in a murine model of LD, IL-10 and miR-155 have opposite effects on the development of carditis, although both were required for the suppression of carditis and MiR-155 had little effect on arthritis [77]. Certain murine strains (usually derived from *Mus musculus domesticus*) are susceptible to infection and arthritis, whereas wild *P. leucopus* is unaffected. Susceptibility is defined by genetic factors, like major histocompatibility complex (MHC) types, susceptibility to lupus (NZB/W, MRL/*lpr*, and BXSB/*Yaa*), and induced models of human disease-like collagen-induced arthritis, pristane-induced lupus, and bleomycin-induced scleroderma.

The “flu-like syndrome” of early LD is likely due to the release of inflammatory mediators, similar to that seen in viral infections. The recurrent and often intermittent “flu-like symptoms” reported by some patients during their courses of “chronic LD” are likely non-specific, not related to *B. burgdorferi* infection, and not immunologically mediated, since intermittent cytokine release, e.g., IL-1, IFN-γ, with no contemporaneous intermittent immune stimulus is unlikely.

The production of pro-inflammatory cytokines may occur when debris activates immune cells. The persistence of organism-derived debris may drive inflammation in reactive arthritis [78]. Antibiotic therapy may prevent the chronic synovitis of LD in keeping with the premise that early synovitis is due to active local infection, but later antibiotic-resistant Lyme arthritis is not. An antibody pattern in synovial fluid differentiating antibiotic-responsive from refractory Lyme arthritis has been described [79], as has a pattern of T cell antigen receptors in antibiotic-resistant Lyme arthritis [80], with the latter mapping primarily to T peripheral helper (Tph) cells as opposed to classical Th1 cells. If substantiated, these findings may aid in future clinical decision-making.

**Autonomous self-perpetuating immune/inflammatory reaction**

Early studies suggested Lyme arthritis was driven by the Th1 cell response with IFN-γ predominance [81]. More recently, a fundamental role for Th17 cells has been identified, summarized by Nardelli, Callister, and Schell [82]. The autonomous activation of Th17 without further antigen-specific mechanisms may explain antibiotic-refractory arthritis in the absence of ongoing infection [83,84]. IL-23 may play a role in murine Lyme arthritis [85]. Lyme arthritis may involve the dysfunction of regulatory T cells (Treg; CD4+CD25+ T cells) [86,87], perhaps explaining the non-resolution of patients’ Lyme arthritis [75]. Means by which a self-perpetuating disease might develop after infection were summarized by Singh and Girschick, including molecular mimicry; chronic poorly controlled inflammation; Th1 cytokines and other gene products, including IL-1, TNFα, collagenase, and prostaglandins elicited from macrophages; and osteoclast activation by IL-1 and TNFα [88]. Lyme synovitis fluid T cells from expressed high levels of CD45RO, the memory cell marker compatible with prior activation. Skewing of the TCR was found, similar to findings by this group in RA and in psoriatic skin lesions. The stimulation of synovial fluid T cells by *B. burgdorferi* resulted in proliferation but no increase in an individual TCR. The authors proposed this suggested skewing of the TCR repertoire of fresh Lyme synovitis fluid T cells representing a synoviotropic or nonspecific inflammatory response rather than an antigen-specific response to the organism [89].

**Molecular mimicry**

“Molecular mimicry” occurs when a foreign immunogenic molecule, as determined by the host’s immune system, resembles a host molecule, then eliciting an immune response targeting the host’s “cross-reacting” epitope (the precise region of the antigenic molecule bound by the antigen-binding region of the antibody and/or TCR), which can damage the host. Such a phenomenon occurs in Chagas disease—*Trypanosoma cruzi* cross-reacts with human cardiac muscle and peripheral nerve, causing Chagasic cardiomyopathy and neuropathy, respectively, and rheumatic fever—M proteins of certain Group A β hemolytic Streptococci cross-react with human cardiac myosin, causing rheumatic carditis.

Cross-reactivity between *B. burgdorferi* and synovial antigens has been proposed to explain the highly localized focus of inflammation, which is in the joints. Steere and colleagues found that an epitope of lymphocyte function-associated antigen 1 (LFA-1; also known as integrin αLβ2 and CD11a/CD18) cross-reacted with *B. burgdorferi*’s outer surface protein A (OspA) (31 kDa) [90]; both epitopes are presented by the same HLA-DR molecule, HLA-DR4. HLA-DR4 had previously been associated with antibiotic-refractory arthritis and was implicated as a risk factor. The same group found that antibiotic-refractory arthritis was associated with T cell responses to OspA epitopes, although not the cross-reacting epitope [91,92]. This and further work suggested to the authors that molecular mimicry, with organ-specific autoimmunity, underlay antibiotic-refractory arthritis [93,94,95,96,97]. Most patients with Lyme arthritis have increased frequencies of T cells reactive with the OspA161–175 peptide, demonstrating a decrease in these antigen-specific Lyme synovitis fluid T cells in following antibiotic therapy regardless of the response to antibiotics. The lack of correlation might be interpreted as indicating that these cells are not the cause of the refractory synovitis [98].

Kalish et al. found no association between clinical status and T cell responses to OspA or LFA-1 [99]. Maier and colleagues found that OspA-specific T cells recognized multiple epitopes on many other human proteins, leading them to conclude “…the existence of cross-reactive epitopes alone does not imply molecular mimicry-mediated pathology and autoimmunity” [100].

Furthermore, in neither relevant mouse strains nor humans was OspA vaccination associated with the onset of arthritis [101,102]. LFA-1 is a ubiquitous protein, so systemic auto-immunity would be expected. Furthermore, arthritis in a larger number of joints might be expected. One might speculate that the cross-reactivity was present only in the joint, perhaps only in a joint previously damaged, and therefore revealing the specific LFA-1 epitope OspA reactivity binds other cross-reacting epitopes on other proteins, e.g., mitogen-activated protein kinase activator with WD40 repeat-binding protein (MAWD-BP) and cytokeratin 10 [103]. OspA and the M5 protein of *S. pyogenes* share significant DNA homologies [104]. This was accomplished using an ELISA and immunoblot antibodies on the M5 protein and sera from *B. burgdorferi*-infected mice that reacted with OspA and human myosin. NZB mice, often a good model for autoimmune diseases, infected with *B. burgdorferi* developed greater joint swelling and higher levels of anti-*B. burgdorferi* cross-reactive IgM than other mouse strains with the same major histocompatibility complex loci (DBA/2 and BALB/c).

Another example of molecular mimicry is found between a linear epitope of *B. burgdorferi*’s flagellin (p41) (41 kDa) and one of human heat shock protein 60 (HSP60) (60 kDa), an epitope exposed only in the cytoplasm, not mitochondria [59,105,106,107,108]. H9724, a monoclonal antibody to flagellin, binds both epitopes. Sera from patients with LD neurologic manifestations bound to nerve fiber sections and to these two epitopes. H9724 penetrated neuroblastoma cells in culture, profoundly suppressing the outgrowth of dendrites [109]; the cytoplasmic extensions from neuroblastoma cell bodies mimicking outgrowths are meant to receive electric signaling from other neural cells. It is unlikely that the alteration of axonal–neuronal function is a cause of the patchy peripheral neuropathy of LD, which is antibiotic-sensitive and implicates infection. There would seem to be no clinical role for this cross-reactivity.

Up to 30% of patients with chronic Lyme arthritis have antibodies to endothelial cell growth factor (ECGF), an IFN-inducible signaling protein, and it is found at higher levels in the synovial fluid of antibiotic-refractory arthritis than in those with antibiotic-sensitive disease. This correlates with findings of obliterative microvascular lesions. ECGF stimulates endothelial cell growth and proliferation, enhancing new blood vessel growth. These autoantibodies may also be found in early infection months before the synovitis [110], although there is no evidence that ECGF reactivity results from molecular mimicry. MMP [111] and four novel HLA-DR-presenting peptide autoantigens, presented in synovial tissue and/or PBMCs [112], are also recognized in the immune response to *B. burgdorferi*. Another study showed that CD4+ T cells are reactive with at least one epitope of three extracellular matrix proteins, fibronectin-1, laminin B2, and/or collagen Vα1, suggesting to the authors that this is an example of epitope spreading rather than molecular mimicry, and it has not been established as a cause but rather the result of chronic synovitis [113].

Over 13 years, Arvikar et al. collected a series of 30 patients with a “new onset systemic autoimmune joint disorder, a median of 4 months after Lyme disease”. Of these 30 patients, 15 had rheumatoid arthritis, 13 psoriatic arthritis, and 2 peripheral spondyloarthritis. Many had “Lyme disease-associated autoantibodies” at MMP-10, ECGF, and apolipoprotein [Apo]-B100. The authors offer three plausible explanations for this association, namely (1) pure coincidence, (2) induction by a non-specific adjuvant effect from a component of the organism, or (3) an auto-immune reaction that specifically induced *B. burgdorferi*. Therapy for such patients should be the disease-modifying agents used in RA [114]. These two examples of molecular mimicry are of theoretical interest, although neither is of proven clinical relevance. Others have found a low prevalence of autoantibodies, including Ro52, Scl70, and anti-fibrillarin, although the relevance of ongoing clinical problems remains unclear [115]. Danner et al., in their studies of murine adaptive T and B cell responses in Lyme arthritis, found that peptides derived from human Lyme autoantigen apolipoprotein B-100 (apoB-100) were enriched in infected Il10^−/−^ mice (with evidence of epitope expansion), while the B6 genetic background enriched for responses to peptides were derived from neutrophil extracellular nets [116].

It is said that Archimedes claimed that given a long enough lever arm, he could move the world. Using computerized searches, targeted peptide synthesis, liquid chromatography, mass spectrometry, and other techniques, one can find high-stringency cross-reactivity between pathogen-related molecules and many human proteins in data banks. One must separate the wheat from the chaff.

Serum immune complexes [117,118,119] and cryoglobulins [120,121,122] have been found in patients with LD, but these complexes are not of proven immunopathogenic relevance; no vasculitic or immune complex-driven syndromes have been described in LD.

In summary, the interaction between *B. burgdorferi*, a complicated organism with many identified mechanisms to alter, subvert, and avoid the human innate systems, and acquired immune responses, ultimately triggers host immune responses. In some patients, this is rapid and robust, and there are no clinical sequelae. In others, persistence and dissemination with local (EM) and distant inflammatory responses occur, causing disease. As there is no evidence that this organism produces toxins, the tissue damage and disruption of function is due to the host immune responses, which are cellular and humoral and innate and acquired. Undoubtedly, more undetected interactions between *B. burgdorferi* and the human host will be described, enhancing our understanding of the immunopathogenesis of and treatment for Lyme disease. Assisting our understanding of the post-infectious findings in some patients would be the development of a rigorous classification system [115,116,123,124,125,126,127,128,129,130,131,132,133,134,135].

## Data Availability

No new data were created or analyzed in this study.
